# The nexus of natural killer cells and melanoma tumor microenvironment: crosstalk, chemotherapeutic potential, and innovative NK cell-based therapeutic strategies

**DOI:** 10.1186/s12935-023-03134-y

**Published:** 2023-12-06

**Authors:** Azadeh Rahimi, Zahra Malakoutikhah, Ilnaz Rahimmanesh, Gordon A. Ferns, Reza Nedaeinia, Sayed Mohammad Matin Ishaghi, Nasim Dana, Shaghayegh Haghjooy Javanmard

**Affiliations:** 1 Applied Physiology Research Center, Cardiovascular Research Institute, Isfahan University of Medical Sciences, Isfahan, Iran; 2 Division of Medical Education, Brighton and Sussex Medical School, Falmer, Brighton, Sussex, BN1 9PH UK; 3 Pediatric Inherited Diseases Research Center, Research Institute for Primordial Prevention of Non-Communicable Disease, Isfahan University of Medical Sciences, Isfahan, Iran; 4 Department of Microbiology, Faculty of biological science and technology, University of Isfahan, Isfahan, Iran

**Keywords:** Melanoma, Metastasis, Natural killer (NK) cells, Tumor microenvironment

## Abstract

The metastasis of melanoma cells to regional lymph nodes and distant sites is an important contributor to cancer-related morbidity and mortality among patients with melanoma. This intricate process entails dynamic interactions involving tumor cells, cellular constituents, and non-cellular elements within the microenvironment. Moreover, both microenvironmental and systemic factors regulate the metastatic progression. Central to immunosurveillance for tumor cells are natural killer (NK) cells, prominent effectors of the innate immune system with potent antitumor and antimetastatic capabilities. Recognizing their pivotal role, contemporary immunotherapeutic strategies are actively integrating NK cells to combat metastatic tumors. Thus, a meticulous exploration of the interplay between metastatic melanoma and NK cells along the metastatic cascade is important. Given the critical involvement of NK cells within the melanoma tumor microenvironment, this comprehensive review illuminates the intricate relationship between components of the melanoma tumor microenvironment and NK cells, delineating their multifaceted roles. By shedding light on these critical aspects, this review advocates for a deeper understanding of NK cell dynamics within the melanoma context, driving forward transformative strategies to combat this cancer.

## Introduction

Melanoma is the type of skin cancer with a high mortality because of its high propensity to metastasize [1]. However, the molecular mechanisms that enable melanoma cells to colonize distant organs and their interactions with the metastatic niche are still poorly understood [2]. After conducting a significant amount of research over the past decade, it has been determined that the tumor microenvironment (TME) does not solely receive immune cells passively, but rather actively participates in the advancement of immunosuppressive conditions, which in turn enhances the spread of melanoma cells to distant organs. Whilst the metastatic niche may initially be hostile to the colonization of cancer cells; tumor-derived signals can shift the balance towards a metastasis-favoring condition, leading to the development of a hospitable TME that promotes tumor growth and dissemination [2].

The interaction of melanoma and immune cells is a major contributor to tumor progression and metastasis, which is defined in three distinct phases. During the elimination phase, the immune system efficiently eliminates transformed cells, thereby preventing tumor initiation. Next, the emergence of “immune-resistant” cells through the acquisition of favorable mutations and subsequent properties leads to the equilibrium phase. However, the host immune system is still able to control tumor growth by killing immune-sensitive cells. The final stage is distinguished by immune evasion, where melanomas use various tactics to suppress and evade recognition and elimination by both innate and adaptive immunity [3, 4].

The high mutational burden of malignant melanoma makes it one of the most immunogenic cancers; oncogenes commonly mutated in melanoma induce the expression of cytokines, chemokines, enzymes, and growth factors, which recruit and regulate immune cells [1, 5]. Melanoma cells have a great degree of plasticity, which allows them to quickly develop escape mechanisms that allows adjustment to an unfavorable TME. Both the ability to release immunomodulatory compounds and the ability to develop a less immunogenic phenotype are present in tumor cells. Additionally, regardless of whether a cell has transformed, all other TME cells can to adapt to the hostile TME and produce immune-modulatory signals or mediators that affect immune cell activity directly or indirectly by stimulating other cells in the tumor site [6]. These properties can further affect the composition and recruitment of immune cells or other infiltrates within the TME, in favor of metastasis [2].

Hence, in recent years, the manipulation of the immune system has emerged as one of the most important therapeutic strategies for melanoma. Numerous studies have provided evidence in favor of the idea that innate immunity, the body’s initial line of defense against infections and tumors, plays a significant role in the genesis, progression, and prognosis of melanoma. Therefore, the best possible stimulation of innate immune cell populations may restore functioning immune responses and slow tumor growth [7, 8]. The first immune cells that participate in non-specific immediate cytotoxicity towards melanoma cells are tumor-resident macrophages, polymorphonuclear neutrophils (PMN), NK, and dendritic cells [6].

NK cells, which are skin-resident innate cytotoxic lymphocytes, can eradicate target cells on their own without prior sensitization. Through germline-encoded receptors that are expressed on the target cells, they identify cells that need to be eliminated. Such ligands, which are recognized by the inhibitory or activating receptors, can have either inhibitory or activating effects. Because of this, the balance of signals sent by various receptors determines whether NK cells will activate or die [6, 9].

The initial immune response against melanoma is significantly dependent on the contribution of natural killer cells, which not only interact with dendritic cells but also secrete cytokines to facilitate the development of an appropriate adaptive immune response [10].

In vitro experiments have conclusively demonstrated that NK cells possess the ability to identify and eliminate melanoma cells [11–13]. The efficacy of NK cells in combating melanoma in vivo has been established through animal models [14], and the observation of changes in natural killer cells, such as the reduction of activating receptors or exhaustion, in patients with melanoma provides additional evidence to support this idea [15, 16]. These observations suggest that melanoma cells may have developed mechanisms to evade NK cell-mediated.

In this review, we examine the connection between the components of the melanoma tumor microenvironment and the significant role played by natural killer cells in controlling melanoma metastasis. We also present potential therapeutic strategies to utilize natural killer cells for the prevention or treatment of this cancer, given their influential role in the melanoma tumor microenvironment.

## Relationship between melanoma and tumor microenvironment components

The tumor microenvironment is a highly complex and dynamic ensemble of cells that influences cancer cell behavior and can define cancer initiation, growth, development, and multidrug resistance [17].

**Fig. 1:**
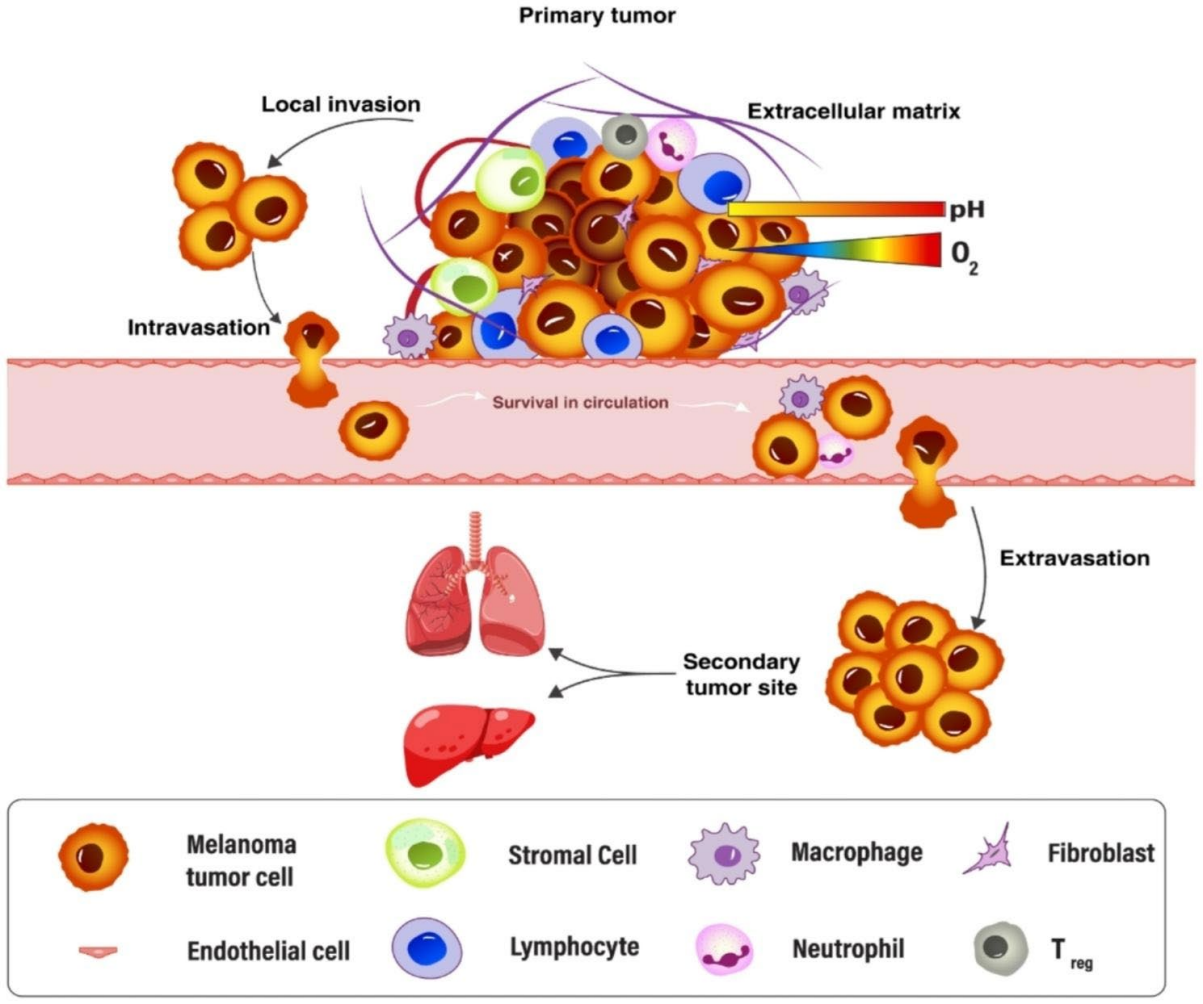
This figure illustrates the relationship between the tumor microenvironment (TME) and the progression of melanoma cancer. The TME is composed of various cell types, such as cancer-associated fibroblasts (CAFs), blood endothelial cells, immune cells, and the extracellular matrix (ECM)

The TME consists of cancer cells and various types of tumor stromal cells, including stromal fibroblasts, endothelial cells, and immune cells such as microglia, macrophages, and lymphocytes. Additionally, non-cellular extracellular matrix components such as collagen, fibronectin, hyaluronan, and laminin are also present in the TME (Fig. 1) [18, 19].

A tumor microenvironment is the collection of cellular and molecular components that provide the setting in which the tumor begins, develops, and finally spreads through normal tissue [20].

Normal epithelial cells; fibroblasts that form the tissue’s supporting structure or stroma; blood vessels that grow in response to tumor signals; resident and infiltrating immune cells; signaling molecules provided by both cancerous and normal cells; and the extracellular matrix (ECM) comprise the tumor microenvironment [17].

The rapid growth of tumor cells and their high metabolic demands place additional needs on cells, creating a highly selective environment known as the tumor microenvironment. This environment includes changes and temporal fluctuations in the biochemical conditions within the tissue, such as hypoxia, low pH, and nutrient deprivation [21].

Melanoma cells have a highly interactive behavior with their surrounding environment, which involves not only direct interactions with other cells and the extracellular matrix but also the release of growth factors and cytokines into the surrounding area [22]. For melanoma cells to successfully settle in a new location during the initial stages of invasion, they need to initiate a mechanism that enables them to move, infiltrate, and survive in an unfamiliar environment with varying microenvironmental conditions. Melanomas trigger growth factor loops that govern cell sticky qualities, allowing them to survive in otherwise unfavorable environmental settings [23].

The tumor microenvironment has long been recognized as an active participant in carcinogenesis. Stromal makeup differs from tumor to tumor. In cutaneous melanoma, the stroma is either desmoplastic (fibroblasts and fibrocytes with considerable fibrillar ECM component accumulation) or myxoid (atypical spindle cells with significant proteoglycan accumulation) [24]. The main ECM component in the dermis is collagen I, which is generated primarily by fibroblasts.

Up to 80% of the melanoma tumor mass may be comprised of fibroblasts [25], and normal fibroblasts can inhibit melanoma growth by the paracrine attraction of immune cells [26]. However, melanoma secretes several substances, including transforming growth factor-beta (TGF-β) and Nodal (a TGF superfamily member), which induce the transformation of normal fibroblasts into protumorigenic cancer-associated fibroblasts (CAFs) with a myofibroblast phenotype [27–30].

The number of CAFs within the tumor population may increase as melanoma progresses, and they may exhibit a variety of functions, including immunosuppression caused by the activity of TGF-β [31]. These cells may affect the development of melanoma in several ways, such as by producing matrix metalloproteinases that promote melanoma invasion [32]. Furthermore, CAFs secrete vascular endothelial growth factor (VEGF) and multiple chemokines in the surrounding tumor sites, promote angiogenesis, and actively recruit endothelial progenitor cells to the tumor site [25].

Activated fibroblasts impact on the resistance of melanoma treatment by producing various growth factors. CAFs have diverse functions in the development, metastasis, and resistance to medication in melanoma. These functions are influenced by intercellular communication, the release of extracellular matrix components, growth factors, and cytokines [32, 33].

Lymphocytes (including NK cells, T cells, and B cells), mast cells, myeloid-derived suppressor cells, dendritic cells, and macrophages are among the immune cells found in the tumor microenvironment [34].

Melanoma cells hinder the development of dendritic cells, thereby allowing them to evade the complex process of T-cell activation. These tumor cells secrete inhibitory cytokines which impede dendritic cell maturation. Consequently, this disease impairs the ability of dendritic cells to present antigens to T cells, leading to a weakened immune response [35]. Additionally, patients with melanoma experience reduced dendritic cell counts, which has been associated with a poor prognosis [36]. DC subsets regulate T-lymphocyte function and impact the clinical outcome of melanoma [37]. A lower count of DCs is associated with metastatic melanoma, while a higher count indicates no metastasis or a low risk of recurrence [38]. Overall, the function of DCs plays a crucial role in determining the prognosis of melanoma.

Dendritic cells are activated through Toll-like receptors (TLRs), recognizing the molecular characteristics of potential pathogens [39].TLRs are an important family of receptors of the innate immune system responsible for detecting the conserved molecular patterns exhibited by pathogens [40]. Notably, recent investigations have revealed that the activation of TLR-4 induces a substantial increase in the expression of pro-inflammatory and immunosuppressive cytokines, as well as inflammatory factors, when specific ligands are applied to melanoma cells [41, 42]. Proinflammatory cytokines and type I IFNs are produced by DC in response to TLR ligands [37]. Additionally, studies indicate that tumor-derived TLR2 ligands, stimulating TLR2^+^ DCs, impair the activity of DCs in mice melanoma [39]. DC modulation affects both tumor development and anti-melanoma immunity.

As melanoma progresses, the number of other immune cells and neutrophils infiltrating the tumor increases [43]. Neutrophils can be divided into two populations: the N1 subtype, which is the dominant group in the early melanoma microenvironment and shows anti-tumor effects, and the N2 subtype, which emerges in later stages and exerts immunosuppressive effects [44]. In contrast, mast cells (MCs), which are long-lived tissue-resident cells abundant in human skin, are associated with melanoma stroma (MAMCs). Due to their pro-tumorigenic function and influence on melanoma development and metastasis, mast cells have shown diverse effects on melanoma [45]. Mast cells contribute to the growth of melanoma by reacting to substance P-induced neurogenic inflammation [46]. Consequently, mast cells release a variety of cytokines, proteases, and biological factors that weaken antitumor defenses [47].

Other extracellular components and cells in the tumor niche, such as keratinocytes, miRNAs or exosomes, and adipose tissue, can also influence the unique immune response in the TME [48]. MiRNAs and small non-coding RNAs that regulate protein translation attenuation or suppression play a role in modulating the immune microenvironment of melanoma [49]. As mentioned above, different types of lymphocytes play important roles in melanoma. B lymphocytes are the cells in charge of acquired and humoral immunity. Although one of their secondary functions is to retain immunological memory, their major function is to mediate the production of antigen-specific immunoglobulins [50]. These professional antigen-presenting cells (APCs) play a wide range of roles in melanoma [51].

The development of resistance to targeted therapy is facilitated by tumor-associated B cells (TAB), which account for more than 33% of the TME immune cells in melanoma. TABS also promote angiogenesis and persistent inflammation. Furthermore, melanoma cell metastasis is increased and overall patient survival is decreased when B lymphocytes are present in the tumor infiltrate [52].

Several studies have presented conflicting results concerning the activation of these cells within the melanoma tumor microenvironment. Recent studies on melanoma have indicated that non-metastatic melanoma is distinguished by a high density of B-cells, which correlates with a better prognosis. However, other studies have discovered that melanoma cells generate fibroblast growth factor 2 (FGF2), which stimulates B-cells to produce insulin-like growth factor 1. This, in turn, could potentially lead to resistance against BRAF and MEK inhibitors [52, 53]. However, tumor-associated B cells may function in a reverse manner in melanoma in response to immunotherapy. A particular TAB subtype can make it easier for CD8 ^+^ T lymphocytes to enter the tumor compartment, which can improve melanoma response to ICIs. According to scientists, pretreated melanoma patients with larger quantities of these B cells respond better to subsequent immunotherapy therapies [54].

The other types of lymphocytes are T cells that identify antigenic peptides that are presented by other immune system elements that make up the immuno-microenvironment [55]. Through the release of certain cytokines, CD4^+^ T cells serve as immune response “adjuvants”. In contrast, CD8^+^ T lymphocytes are directly involved in antigen/tumor cell differentiation and elimination [56]. A recent study of metastatic melanoma samples revealed that the presence of tumor-associated CD8^+^ T cells and CD20^+^ B cells is associated with increased survival [57]. In CD8^+^/CD20^+^ tumors, the development of tertiary lymphoid structures is associated with a gene profile that forecasts melanoma patient-reported outcomes using immune checkpoint inhibitors (ICIs).

A subset of T lymphocytes with elevated concentrations of the inhibitory receptor PD-1 and T-cell immunoglobulin domain and mucin domain 3 (TIM3) was found in patients with advanced melanoma. TIM3 inhibition improved T cells’ antitumor efficiency and partially reversed their dysfunctional state. These findings justify concurrent PD-1 and Tim3 inhibition as a potential treatment strategy to restore CD8 ^+^ T cell activity in melanoma [58]. In a recent analysis of tumor infiltrate in melanoma, it was discovered that the defective CD8 ^+^ T cell subgroup exhibited significant clonal expansion. The authors highlighted the importance of understanding the differentiation and reactivity of these cells, as it is probable that they have an important function in controlling the anti-cancer activity and resistance to immunotherapeutic drugs. Consequently, these cells become an appealing objective for immunotherapeutic treatments that are more precise and efficient in treating melanoma [59].

The third group of lymphocytes, known as natural killer cells, can be categorized into two main subgroups based on the expression of CD56 and CD16. Certain natural cytotoxicity receptors (NCRs) play a crucial role in facilitating tumor destruction by NK cells [60]. Notably, NKp30, NKp44, and Natural-Killer Group 2 Member D (NKG2D) are the main receptors responsible for activating NK cells’ anticancer properties. However, it is worth noting that the expression of these receptors can be suppressed by various factors present in melanoma TME [61].

The TME’s NK cells can directly lyse tumor cells, which is just one of their many effects on tumor cells. The buildup of activating signals that outweigh the inhibitory signals triggers NK cell-mediated cytotoxicity. During the malignant transformation process, many cancers exhibit increased expression of ligands for NK cell-activating receptors. Despite this, numerous types of cancer, including melanoma, have developed tactics to evade detection and elimination by NK cells [62]. There are various ways in which NK cells can be avoided by melanoma cells, such as through direct interaction with tumor cells, secretion of cytokines or molecules by tumor cells or immune-suppressive cells, and the creation of a hypoxic environment within the tumor [10]. The survival rate of melanoma in mouse models was found to decrease significantly when NK or CD8 T cells were depleted, despite treatment with anti-PD-1 anti-CTLA-4. This suggests that NK and CD8 T cells work together to eliminate the tumor in response to these therapies [63]. Patients with advanced melanoma receiving anti-PD-1 therapy have significantly more CD16 NK cells [64].

NK cells occupy a crucial position between the innate and adaptive immune systems and have significant functions in the melanoma tumor microenvironment. In the following sections, we will examine the important role of NK cells in managing melanoma metastasis and suggest potential therapeutic methods to utilize NK cells for preventing or treating this cancer.

## The significance of natural killer cells in recognizing melanoma

**Fig. 2:**
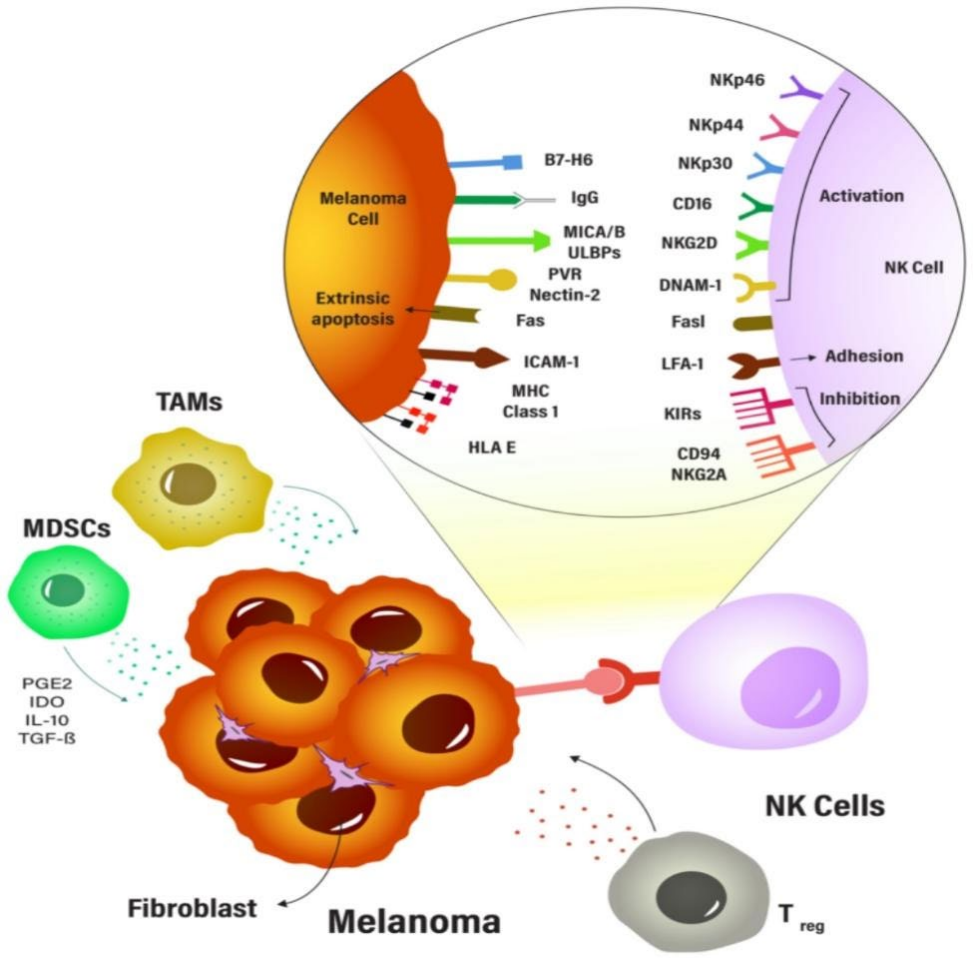
The tumor microenvironment can affect the activity of NK cells in melanoma. The presence of immunosuppressive immune cells in the TME can directly impact the function of NK cells. Therefore, understanding the complex interactions between immune cells and NK cells in the TME is crucial for developing effective immunotherapies for melanoma

More than forty years ago, tumor-related effects on the activity of natural killer cells in melanoma patients were reported for the first time [65, 66]. Recognition of melanoma cells by NK cells occurs following diverse ligand-receptor interactions, particularly as research has demonstrated that melanoma cells express diverse ligands for various NK cell activation receptors. Generally, NK cell activities are dynamically controlled by the interaction of activating and inhibiting signals. The abundance of ligands for the NK cell activation receptors increases only during situations of cellular stress, such as viral infection or the onset of malignancy [67]. On the other hand, major histocompatibility complex (MHC) class I molecules or human leukocyte antigen (HLA) class I, typically expressed on healthy nucleated cells, provide inhibitory signals to NK cells cytotoxicity in a normal setting by interacting with a variety of killer cell immunoglobulin-like receptors (KIRs) and/or Natural Killer Group 2 A/ CD94 (NKG2A/CD94) [1, 6]. KIRs are the most known important inhibitory receptors found on NK cells, which belong to a family of receptors with a high degree of polymorphism and are capable of recognizing MHC class I molecules. Significantly, only a certain subset of NK cells express each form of KIR [68, 69]. Even though most KIRs are inhibitory receptors, some of them are activating receptors, which are thought to have evolved from inhibitory KIRs [70]. KIRs commonly interact with HLA types A, B, or C, whereas HLA type E usually binds to the NKG2A/CD94 inhibitory receptor (Fig. 2) [1, 6]. MHC I molecules are inadequately expressed whenever melanomas progress.

Natural cytotoxicity receptors ,and NKG2D, and DNAX accessory molecule-1 (DNAM-1) are important receptors that are involved in the activation of NK cells against melanoma [62]. NCRs play a key role in the elimination of tumors by NK cells. NK cells in humans express the NCRs including NKp46, NKp80, and NKp30; however, NKp44 is increased following the activation of certain NK cells by interleukin-2 [71, 72]. It was found that melanoma cells hindered the immune function of natural killer (NK) cells by suppressing the expression of key NK receptors such as NKp30, NKp44, and NKG2D. This inhibition led to a decline in the NK cell’s ability to destroy different types of melanoma cell lines [73]. Additionally the surface density of NCR controls the ability of NK cells to kill susceptible target cells [74]. Ligands of NCRs can also be impacted by tumor heterogeneity and anatomical origin in metastatic melanoma. For instance, NKp30 ligands had a very low level of expression in metastatic melanoma, whereas NKp44 and NKp46 ligands had variable levels of expression [75]. Another NK cell activation receptor, NKG2D, has been discovered to be associated with NK cell degranulation and Interferon-gamma (IFN-γ) expression [15].

NKG2D can recognize two different MHC-like molecules, the MHC class I chain-associated proteins (MIC) A and B, as well as unique long 16-binding proteins (ULBPs) induced by stresses like malignant transformation [76, 77]. After examining different melanoma cells, it was discovered that MICA/B and ULBPs are extensively present in melanoma cells [13]. DNAM-1 (also called CD226) is a transmembrane protein whose expression has been reported different in NK cells [78]. Both CD112 (Nectin-2), as well as CD155 (poliovirus receptor, PVR), which are widely expressed by cancer cells such as melanoma, are recognized by DNAM-1 [13, 79]. By attaching to the Fc component of immunoglobulins IgG1, IgG3, IgG2, and IgG4 in NK cells, CD16a functions as a prototype NK cell-activating receptor. By facilitating antibody-dependent cell cytotoxicity (ADCC), CD16 helps kill melanoma cells using the engagement of CD16a to IgG, in contrast to other activating NK cell receptors. Tumor cells are killed when CD16a binds to IgG, causing NK cells to produce perforin and granzyme B [62].

NKG2A the other significant NK cell inhibitory receptor, builds heterodimers with CD94 and identifies HLA-E [80]. Although HLA-E is either absent or just weakly expressed in melanoma, the presence of NK cells followed by a high Interferon-gamma response is likely what increases HLA-E in melanoma cells [81]. Activating and inhibiting NK cell receptors in melanoma cells that have been reported are summarised in the Table 1.

**Table 1:** Major NK cell receptors in melanoma

Receptor	Molecular structure	Expression	Sample type	Function
NKp 30 (NCR)	Immunoglobulin Superfamily	Resting and activated NK cells	stage IV melanoma patients	Activator [75]
NKp 46 (NCR)	Immunoglobulin Superfamily	Resting and activated NK cells	stage IV melanoma patients	Activator [75]
NKp 44(NCR)	Immunoglobulin Superfamily	Activated NK cells	1106mel melanoma cell line	Activator [82]
NKp 80 (NCR)	Immunoglobulin Superfamily	Resting and activated NK cells	melanoma metastasis patients	Activator [83]
NKG2D	C-type lectins	NK, gamma delta T, CD8^+^ T cells	stage III–IV melanoma patients	Activator [1]
DNAM-1(CD226)	Immunoglobulin Superfamily	All NK cells, T cells and monocytes	B16F10 melanoma cell line	Activator [84]
KIR	Immunoglobulin Superfamily	NK, T cells	stage I–IV melanoma patients/ melanoma cell line	Inhibitor/ Activator [62]
NKG2A/CD94	C-type lectins	NK, Cytotoxic T-lymphocyte	B16F10 melanoma cell line	Inhibitor [85]

## Presence of natural killer cells in the tumor microenvironment of melanoma

The importance of NK cells in the melanoma microenvironment is attributed to the interaction of ligands and cell surface receptors with other TME cells, particularly melanoma cells. Cellular stress induces the recruitment of NK cell-activation receptors. The transformed melanoma cells in the early stage significantly decreased the expression of HLA molecules in such a way that it moved the balance toward the activation of NK cells against these cells. However, melanoma cells with the aid of other TME components (cells, factors, and stroma) can dampen the activation receptors and upregulate the inhibitory receptors on NK cells, thereby promoting tumor progression. These changes include a marked reduction in NKp46 expression in blood NK cells from patients with stage IV metastatic melanoma as well as a change in their functional capabilities [1, 86].

Although NK cells are found in lymph nodes draining a tumor, they are usually absent in primary melanoma lesions. High levels of laminin and collagen type IV in the tumor stroma help in preventing mature and effector NK cell subpopulation from penetrating the tumor’s core. The rate of melanoma metastasis decreases in direct proportion to how effectively NK cells break this protective barrier. However, melanoma metastases lack this tumor escape mechanism and NKs can be found in the center and periphery of tumor metastases [87].

Evidence shows that the transcription of NK cells present in melanoma metastasis differs significantly from the circulating NK cells of the same patients. Most circulating NK cells are characterized by a cluster of cells with a well-defined cytotoxicity gene expression profile. Tumor-infiltrating NK cells, however, show increased variability in their transcriptional states [83].

Tumor-induced biomolecules such as growth factors, cytokines, microRNAs, and exosomes can affect NK cells directly or indirectly through TME interactions [34]. TME key cells like CAFs, Tregs, MDSCs, TAMs, and DCs play pivotal roles in suppressing NK cell cytotoxicity [3]. Cancer cells stimulate CAFs, and the high level of MMPs secretion by CAFs in melanoma TME decreases the MICA/B expression, which prevents the activation of NK cells. CAFs also produce IDO, PGE2, and TGF-β which suppress activating receptors, leading to weakened NK cell-mediated anti-tumor immunity [88]. Recruitment of cyclooxygenase (COX)-2/PGE2 pathway by tumor cells and other cells, such as melanoma-derived fibroblasts, is involved in immune evasion. PGE2 appears to contribute to decreased NK cell activity through the down-regulation of NK-activating receptors, perforins, and granzymes. Moreover, PGE2 also affects NK cell function indirectly by providing an immunosuppressive microenvironment through the induction of Treg cells, macrophages, and MDSCs. Melanoma cells also recruit Tregs to provide immune-tolerant conditions. These lymphocytes inhibit the effector function of NK cells through the secretion of immunosuppressive chemokines including IL-10, IL-35, and TGF-β. Moreover, they can engage DCs through the CTLA-4 pathway to block anti-tumor immune responses [89].

Increased distance from tumor vessels, abnormal tumor vasculature, and high oxygen demand of cancer cells result in hypoxia in TME. Along with genetic mutations, these conditions trigger metabolic reprogramming of melanoma cells, which increases glycolysis. The energy demand for melanoma is primarily based on glycolysis. Overproduction of lactate and protons by glycolytic melanoma cells causes the TME to become more acidic. TME acidification in melanoma helps tumors evade immune responses by affecting the number and function of immune cells such as NK cells. Surprisingly, NK cell suppression can be reversed by the neutralization of the acidic TME [87, 90].

In the presence of hypoxic stress, tumor cells initiate the transcription and secretion of hypoxia-inducible factor 1 alpha (HIF-1α). Nevertheless, HIF-1α is subject to regulation independent of oxygen levels, underscoring the complex interplay between oxygen-dependent and oxygen-independent mechanisms in determining the pathogenesis of melanoma [91, 92]. Within melanoma cells, melanogenesis and its highly reactive intermediates exert a dual effect by amplifying the accumulation of HIF-1α and inducing substantial augmentation of both the HIF-1-dependent and HIF-1-independent pathways. This concerted activation contributes to the progression of melanoma and promotes resistance to immunotherapy [23].

The release of ADAM10 induced by HIF-1α results in the cleavage of MICA/B ligands present on the surface of tumor cells. This cleavage leads to the production of soluble MICA/B, which in turn reduces the expression of NKG2D on the surface of NK cells. As a result, the ability of NK cells to eliminate tumor cells is compromised, allowing the tumor to escape from NK-mediated killing. Additionally, hypoxic regions within primary tumors cause the fragmentation of NK cell mitochondria, leading to a decrease in NK cell survival and their capacity to eliminate tumor cells [87].

NK cells try to survive in this hypoxic condition by expression of HIF-1a; however, they fail to express activating NKG2D receptors in response to IL-2 or other activating cytokines (IL-15, IL-12, and IL-21). In response to hypoxia, HIF1a-induced COX overexpression causes increased PGE2 secretion [68]. Hence despite the preserved degranulation level of NK cells as well as the surface expression of other NK cell ligands and receptors, hypoxia reduces the NK cell-mediated killing of melanoma cells. In contrast, melanoma cells up-regulate the HLA-G on the surface, which can directly or indirectly hinder NK cell-mediated killing [6].

NK cells are plastic, and the function of NKs in melanoma depends on the soluble chemicals (such as TGF-β, TNF-α, PGE2, IL-10, IL-12, etc.) in the melanoma TME and the interactions between cells, whether it be between tumor cells or other cells, can cause infiltrating NK cells to undergo dynamic phenotypic changes. This can result in a gradual shift from NK cells that are capable of causing cell death in the early stages to cells that are depleted or have an immunosuppressive effect in the advanced stages (Fig. 2) [6, 93].

### Immunosuppressive properties of the tumor microenvironment on NK cells

In the majority of solid tumors, the response of NK cells is generally ineffective, which could be attributed to two distinct factors. Firstly, the suppressive TME may have a negative impact on the presence of fully competent NK cells at the tumor site. Secondly, the persistence of the tumor may promote immunoediting, further contributing to the ineffectiveness of NK cell responses [94].

In the case of dermal melanoma, as with many other types of cancer, the NK cells present in the TME are known to be functionally impaired. The impairment can be attributed to various factors, including the dysregulation of the activating receptor NKG2D, which has a significant impact on the regulation of cytotoxic activity, cytokine production, and other receptors expressed in NK cells and other lymphocytes [95]. The defective NK cells observed in cancer models have been linked to tumor growth. The development of NK cells throughout the disease can be influenced by both the tumor itself and its surrounding environment [96, 97]. Melanoma cells have stress molecules that stimulate the activation of NK cells, but they also release immunosuppressive factors that affect the expression of different NK receptors responsible for activation [12, 73, 98]. A study was conducted to investigate the relationship between HLA-E molecules and melanoma, revealing elevated levels of soluble HLA-E molecules in stage IV melanoma patients [99, 100].

The NK cells present in the TME of melanoma can eliminate tumor cells directly and/or induce anti-tumoral activities through other immune subpopulations. Nevertheless, the intrinsic pathways of cells within melanoma, as well as their ability to attract and activate suppressive subpopulations, can significantly hinder the cytolytic and anti-proliferative functions of NK cells [9]. In animal models of melanoma, the differentiation of NK cells into mature cells that produce IFN-γ is hindered due to the downregulation of the IL-15 receptor, leading to an accumulation of immature NK cells [101]. Human fibroblasts present in melanoma lesions can also impede the activation of NK cells, substantially diminishing their cytolytic activity [88].

The expression of activating receptors on NK cells can be influenced by human melanoma cells through the modulation of their cognate ligands. Interestingly, it has been observed that overexpression of activating ligands can paradoxically lead to the downregulation of these receptors, and this mechanism has been identified as a major contributing factor [102, 103].

Studies have demonstrated a correlation between elevated levels of the NKG2D ligand ULBP2 in the serum of melanoma patients and larger tumor size, disease progression, and unfavorable prognosis [104]. Moreover, the levels of NKG2D ligands are frequently linked to the stage of the disease, wherein the expression of NKG2D ligands is lower in melanoma metastases compared to primary tumors [105]. According to Konjević et al., [106] the cytotoxic activity of NK cells in patients with metastatic melanoma is impaired due to low expression levels of CD161 and NKG2D.

Additionally, it has been observed that human melanoma cells have the ability to re-express MHC class I molecules, which appears to be influenced by the release of IFN-γ by infiltrating NK cells within the tumor. Notably, melanoma cells that exhibit high levels of MHC class I molecules are found close to the infiltrating NK cells [11].

In conclusion, the TME in solid tumors, including skin melanoma, can have immunosuppressive effects on natural killer NK cells, leading to impaired cytolytic and anti-proliferative functions. The dysregulation of activating receptors, downregulation of IL-15 receptor, and modulation of cognate ligands by melanoma cells are some of the factors that contribute to the ineffectiveness of NK cell responses. Understanding the mechanisms underlying the immunosuppressive properties of the TME on NK cells is crucial for the development of effective immunotherapies for melanoma.

### Effect of chemotherapy on NK cells’ ability to recognize melanoma cells inside TME

Chemotherapy, the primary therapeutic modality employed in the early stages of skin melanoma, yields favorable clinical responses but does not demonstrate substantial efficacy in enhancing overall survival [95]. In vitro and in vivo studies have shown that NK cells can fight melanoma (8), and changes in NK cells, such as a reduced expression/function of NCRs and production of cytokines in patients with melanoma (9, 10), reveal the improvement of escape mechanisms to rescue melanoma cells from NK cell-mediated destruction [15, 107].

Since it has been established that human melanoma cell lines express different ligands for natural cytotoxicity receptors, understanding the interactions between these receptors will help to understand the different stages of melanoma [13]. As melanoma spreads, there is a decreased surface expression of activating ligands, with metastatic lesions expressing these molecules at lower levels than their primary counterparts [12, 108]. NKG2D ligands MICs and ULBPs have received the most attention as ligands shed by melanoma cells, but it has also been reported that B7-H6, which is activated by NKp30, is also shed. As anticipated, shed ligands exhibit a different pattern than their surface counterparts and increase throughout the illness [104, 109]. Contrary to popular belief, overexpression of activating natural killer cell receptors is identified as an important key to reducing NK cell functional responses [102, 110]. Even in epithelia, persistent NKG2D ligand expression impairs NK activity and increases tumor susceptibility throughout the body [110].

NK cells that migrate into tumors have been demonstrated to have diminished NKG2D-dependent and -independent activation pathway functions when studied ex vivo, regardless of their molecular basis. Chronic stimulation of NK cells by tumor cells expressing NKG2D ligands can be used to explain widespread NK cell tolerance. It is essential to determine the elements and circumstances that cause NKG2D function to change from NK cell activation to NK cell tolerance to utilize NKG2D function in tumor immunity [102]. Different investigations have shown that melanoma patients’ NK cells frequently exhibit decreased activating receptor expression, and a diminished ability to react to cancer cells [1, 15, 111].

The activation of the stress response after DNA damage causes cancer cells apoptosis and blocks their proliferation. It can also sensitize tumor cells to elimination by NK cells by enhancing the expression of NKG2D ligands [112]. Chemotherapeutic drugs, such as dacarbazine, temozolomide, and cisplatin, may function against melanoma in part by amplifying the DNA damage response, which causes an increase in NKG2D ligand expression [113]. Dacarbazine mediates the upregulation of NKG2D ligands by melanoma cells that activate NK cells and produce IFN-γ, which leads to an increase in MHC-I presentation by melanomas [114, 115]. It has been suggested that melanoma patients with high levels of NKG2D ligands on tumor cells and activation of NK cells after dacarbazine consumption may respond better to immunomodulation [114].

The antitumor action of both adaptive and innate immunity must be triggered in the tumor microenvironment to effectively inhibit tumor growth. Fregni et al. [86] in their study showed that in patients with metastatic stage IV melanoma, circulating NK cells exhibit distinctive phenotypes and function toward melanoma cells. They observed that patients exhibited changes in NK phenotype and function after chemotherapy. Their findings demonstrate that NK cells participate in the immune response to melanoma and provide fresh experimental justification for their application in immunotherapy regimens for patients with melanoma. In addition, patients with melanoma who received dacarbazine showed an increase in NK cells expressing NKp46, lending credence to this idea [86]. Furthermore, patients who responded to dacarbazine showed increased NK cell cytotoxicity in melanoma cells [116].

Cisplatin has been shown to have a similar ability to increase the expression of NKG2D ligands, which allows it to increase the cytotoxicity of NK cells. In addition, cisplatin increases the expression of other activators such as B7-H6, a ligand expressed by tumor cells, once it interacts with its natural receptor NKp30 and activates NK cell-mediated cytotoxicity [117]. This ligand is only expressed by malignant cells such as melanoma, and not by normal cells [118, 119]. ICAM-1 and Fas are also other activators affected by cisplatin [120, 121]. Furthermore, cisplatin can make cancer cells more susceptible to granzyme B by increasing the penetration of cancer cells and the expression of the granzyme-target caspase-3, which mediates the implementation phase of apoptosis [122, 123].

Different studies have shown that paclitaxel reduces NK cell cytotoxicity by affecting microtubule dynamics [124–126]. Other reports, however, disputed these findings, suggesting that paclitaxel might improve the removal of tumor cells through NK cells by boosting their cytotoxic effects, prompting the expression of ICAM-1 and MIC-B, and/or by making melanoma cells more susceptible to death [122, 126, 127]. These seemingly incongruous results likely depend on the dosages of paclitaxel used in the various studies, as low-dose chemotherapy may induce immunomodulation, whereas high doses are typically thought to be openly immunosuppressive [128].

The immunological effects of docetaxel and paclitaxel are mostly interchangeable. The impact of docetaxel on NK cell-mediated killing varies in studies; some investigators have reported its suppressive effect, while others demonstrated that docetaxel increases NK cell cytotoxicity [126, 129]. The molecular mechanism that has been considered for paclitaxel is that it may increase tumor cell killing by NK cells by enhancing the expression of NKG2D ligands and ICAM-1 [130]. Together, these data show that melanoma chemotherapy drugs can affect the expression of NK cell ligands on melanoma cells and their elimination through NK cells.

### Effect of chemotherapy on NK penetration into melanoma TME

In the TME, before the onset of clinical melanoma and without the need for prior antigen sensitization, NK cells can identify premalignant melanocytes and mount an antitumor response [111]. Additionally, it has been shown that MHC class I molecules are frequently downregulated in both human and mouse melanoma cells, increasing their susceptibility to NK cell-mediated lysis [12, 131, 132]. Upon contact with circulating melanoma cells, NK cells eliminate them [111, 133, 134]. However, their function in the tumor body is severely constrained by their weak permeability to the melanoma tumor microenvironment [135, 136]. Therefore, the ability to permeate melanoma tumors designed to increase NK cell infiltration would be beneficial to take advantage of their inherent cytotoxicity.

It has been shown that alkylating medications affect immune cell infiltration within the TME [137]. Also, they directly increase the susceptibility of melanoma cells to immune cell-mediated clearance [138]. Both DTIC and TMZ have been found to cause melanoma cells to secrete chemokines like CCL5, CXCL9, CXCL10, and CXCL11 in an animal model of melanoma [138].

T lymphocytes express appropriate CCR5 and CXCR3, so the increased chemokine secretion leads to strong infiltration of effector T lymphocytes in the melanoma tumor microenvironment. It has been shown that it correlates with melanoma patients’ outcomes both in terms of survival and tumor response [139, 140]. Since NK cells have these receptors similar to effector T cells, they might be attracted to the melanoma TME via a similar mechanism [141].

Other studies on cisplatin and DTX have shown that the expression of Cisplatin-induced CXCL10 and DTX–induced CXCL11 by melanoma cells affects lymphocyte recruitment and infiltration within tumors [142, 143]. It has been demonstrated that PTX recruits effector cells via a different mechanism. Paclitaxel stimulated lymphocyte relocation and infiltration within the melanoma microenvironment by downregulating L-selectin, a type-I transmembrane glycoprotein that is expressed on leukocytes and NK cells [144, 145].

The structure of the tumor stroma, in particular the compactness of collagen fibers that restrict chemokine accessibility, is another factor affecting lymphocyte infiltration [139]. In this situation, it has been demonstrated that dacarbazine-sensitive melanoma lesions over-expressed genes related to an extracellular matrix organization. This indicates that dacarbanize may activate different pathways that work in concert to promote the efficient recruitment of killer cells [146]. Thus, the alterations in stroma composition brought about by chemotherapy may promote the migration of NK and other cytotoxic cells within the tumor microenvironment, which could help solve one of the major issues with ICI therapy, which is the inadequate infiltration of therapeutic cells [20].

### NK cell-based therapeutic strategies to overcome resistance in Melanoma

Extensive evidence from in vitro and in vivo preclinical studies substantiates the crucial involvement of NK cells in the immune response against tumors. Nevertheless, within the TME, various mechanisms exist that regulate NK cells and hinder their functionality, thereby compromising their ability to exert effective antitumor activities. The combined effects of diminished infiltration of NK cells into the tumor site and the impairment of their functional capabilities contribute to their inability to effectively control tumor growth [95].

The NK cells present in skin melanoma exhibit impaired functionality, contributing to the progression of the tumor. Dysregulation of the activating receptor NKG2D has been identified as one of the underlying mechanisms responsible for the defective state of NK cells. Consequently, therapeutic interventions aimed at reinstating the optimal functioning of NK cells in skin melanoma hold promise as a valuable strategy in the field of tumor immunotherapy [9].

**Fig. 3:**
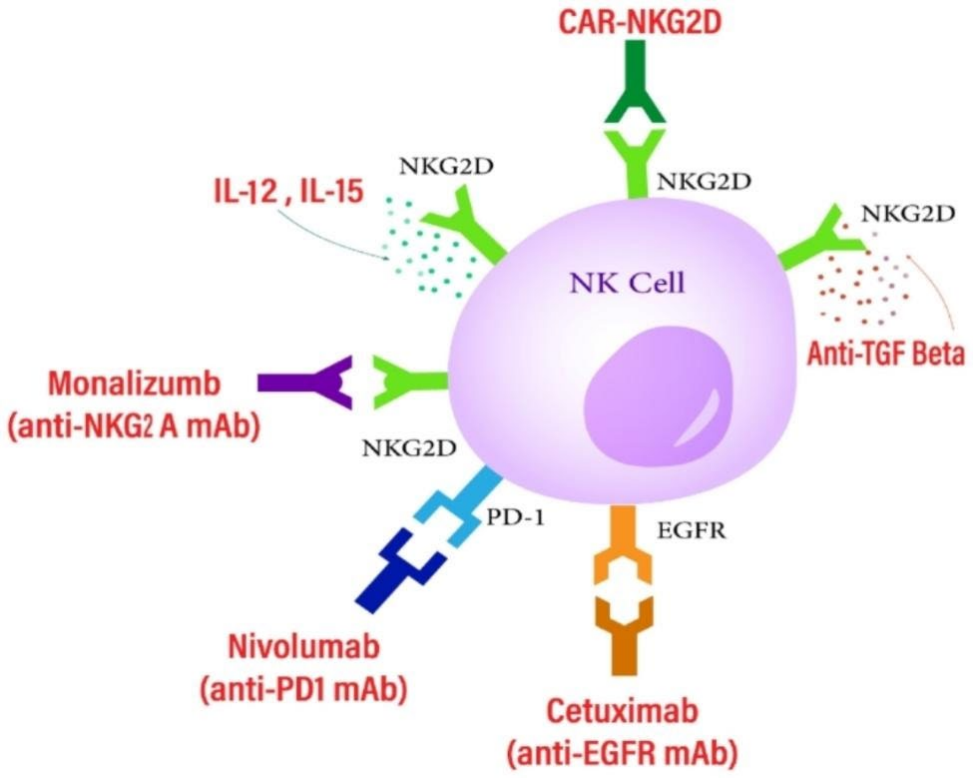
This schematic diagram elucidates the mechanisms by which NK cells can be selectively targeted to exert their cytotoxic effects against melanoma tumor cells, leading to tumor cell death and subsequent regression

Various strategies have been explored to reinstate the functionality of NK cells. One approach involves the activation of NK cells through the administration of cytokines, such as IL-12 and IL-15, which enhance NKG2D expression and signaling, thereby promoting cellular destruction [147, 148]. Another approach involves the genetic engineering of NK cells to express chimeric antigen receptors (CARs) that specifically target NKG2D ligands on melanoma cells, thereby initiating potent cytotoxic responses against the tumor (Fig. 3) [95, 149].

The utilization of antibodies targeting TGF-β can effectively impede resistance mechanisms and reinstate the cytotoxic functionality of NK cells [150]. Additionally, there exists a range of targeted antibodies that can ameliorate the compromised activity of NK cells and augment their antitumor efficacy. The options mentioned include: monalizumab, which targets the inhibitory receptor NKG2A and stops it from suppressing the immune system; nivolumab, which blocks the interaction between the checkpoint receptor PD-1 and the PD-L1 ligand on tumor cells, thus preventing its inhibitory effects; and cetuximab, which specifically targets the EGFR receptor on melanoma cells and stops the transmission of inhibitory signals [95].

PD-1 and T-cell immunoreceptor with Ig and ITIM domains (TIGIT), which are expressed on NK cells, not only play a role in inhibiting NK cell responses but have also been shown to play a role in NK cell instruction. In the absence of TIGIT, NK cells no longer respond to stimuli [151]. TIGIT negatively regulates NK cell function by inhibiting cytotoxicity and IFN-γ production. Blocking TIGIT has been shown to enhance NK cell cytotoxicity and slow tumor growth in a mouse model of melanoma [152]. There has been expanding interest in anti-TIGIT in clinical trials (ClinicalTrials.gov Identifier: NCT02676869, NCT02913313) [62].

Furthermore, antibodies that specifically target NK cell inhibitory receptors, like those that target KIRs, NKG2A, and TIGIT, can boost NK cell responses and subsequently kill tumor cells. Some of these antibodies are currently being tested in clinical trials. Thus, NK cell-based multiple immune combination therapy, based on the pan-specific recognition property of NK cells, is a strategy to further improve the antitumor efficacy [153].

ACT (Adoptive cell therapy) may be a treatment option for people with metastatic melanoma that is resistant to conventional therapies. NK cell therapy has shown a promising clinical safety profile with little toxicity, offering a promising treatment option. NK cells differ from T cells in that they are activated by down- regulation of HLA class I molecules, thereby overcoming the hurdle of tumor immune escape [154].

A decrease in the function of NK cells has, however, been observed in melanoma patients, resulting in a deterioration of the natural defense system. One instance of a clinical need that is largely unmet is in advanced melanoma. The allogeneic cytokine-induced memory-like NK cells (CIML) had potent cytolytic activity and cytokine production toward allogeneic and autologous melanoma target cells, as demonstrated by the authors’ in vitro experiments and use of mass cytometry, indicating that this particular class of NK cell effectors can get around the dysfunction that is frequently noticed in patients with melanoma [155].

Parkhurst et al. [156] studied autologous NK cells to treat patients with melanoma. After non-myeloablative, lymphodepletion chemotherapy, seven patients with melanoma in this study received in vitro activated autologous NK cells. The patients did not exhibit any objective clinical response. The low expression of NKG2D, which is required for persistent NK cells to exert cytotoxic function, was cited by researchers as the cause of this response [156].

The HLA molecules on tumor cells match the KIR molecules on autologous NK cells, so the NK cells don’t get activated because of the lack of alloreactivity. To overcome self-tolerance and achieve the highest levels of NK cell activity, strategies were developed that mismatch the expression of KIR on allogeneic NK cells with that of HLA ligands on tumors. Mismatching KIR-ligands was first applied to hematopoietic transplants [157]. In a subsequent research, Miller and colleagues [158] utilized the same method. They conducted a phase I clinical trial in 2005, where haploidentical allogeneic peripheral blood NK cells were used. The trial involved administering low-dose cyclophosphamide/methylprednisolone and adoptive NK cell transfer to ten patients with metastatic melanoma who participated in the study. After the initial infusion, four subjects showed stable disease states. The second infusion, however, led to a progression of the disease. In the same study, patients pretreated with high doses of cyclophosphamide/fludarabine showed in vivo adoptive NK expansion, suggesting that this high dose of lymphodepletion regimen may be beneficial for patients with melanoma. In addition to peripheral blood, (stem cells from) bone marrow and umbilical cord blood, as well as induced pluripotent stem cells (iPSCs), are additional sources of allogeneic NK cell therapy [159].

It was shown that the CIML NK cells were more effective than traditional NK lymphocytes against transplanted melanoma tumors in a mouse xenograft model [160]. Using NK cell lines as a source for ACT is a great advantage due to their unlimited supply, which is one of their greatest advantages. In a short period of culture, their numbers can be expanded. There is, however, a limitation to their efficacy and persistence because the NK cell lines need to be irradiated before infusion for safety reasons. In 2008, in a phase I clinical trial, Arai et al. [161] used NK-92 cells: Among the melanoma patients, only one responded to the infusion. However, the patient’s condition worsened and he died 255 days after the NK cell infusion.

To overcome the absence of pre-treatment with a lymphodepletion regimen, a high dose of NK-92 cells was required to achieve NK-92 cytotoxicity before the onset of a T-cell immune response. This was accomplished by infusing three doses of NK-92 cells in a short period of five days. It is reasonable to predict that NK cell immunotherapies, in whatever form they may take, will soon play a significant role in the overall oncologic strategy for treating cancer patients [155]. Therefore, it is possible that the use of NK cells in this way can reduce the resistance of melanoma cells and provide more effective treatment based on innate immunity.

### Adoptive transfer CAR-NK cells in melanoma

NK cells can be genetically manipulated to express a chimeric antigen receptor, which specifically recognizes tumor-associated antigens, to improve the targeting of tumor cells [162]. Furthermore, because CARs, unlike TCRs, recognize target antigens independently of MHC recognition and antigen presentation by target cells, they can overcome resistance observed in several malignancies [163].

The chimeric antigen receptor is a fusion protein designed and synthesized that includes several intracellular signaling domains as well as an extracellular antigen recognition region. The single-chain variable fragment (scFv), an extracellular antibody-like component of the CAR that is designed to bind to a particular antigen, and a hinge region with different lengths depending on the location of the epitopes on the target cell [164–166]. In principle, a transmembrane domain (to anchor the chimeric receptor to the membrane of T/NK cells), one or more co-stimulatory domains, and a cytoplasmic signaling domain that induces cytotoxicity as a result of antigen binding are also present in CARs architecture [167, 168].

**Fig. 4:**
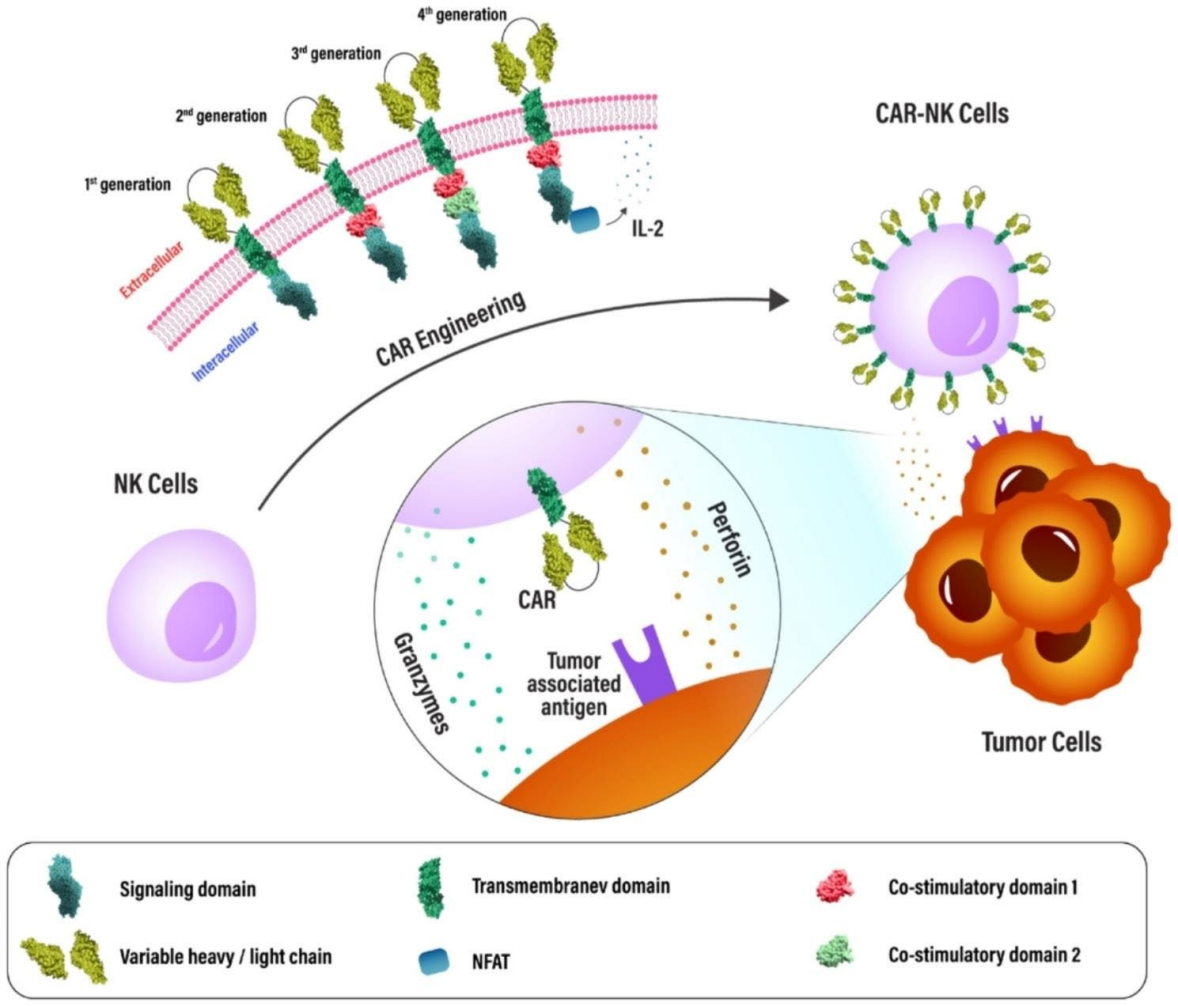
Chimeric antigen receptors (CARs) of the first, second, and third generations, as well as CAR NK cell-based immunotherapy, are depicted schematically. Synthetic extracellular receptors for target antigen recognition, a transmembrane domain, and one intracellular signaling domain are all present in first-generation CAR molecules. For enhanced signaling, second and third-generation CAR constructions are equipped with one or more intracellular co-stimulatory domains. A constitutive or inducible expression cassette for a transgenic protein is a further modification made to fourth generation CARs (also known as TRUCKs (T cells redirected for antigen-unrestricted cytokine-initiated killing)). Upon CAR antigen identification, fourth-generation CARs can activate downstream transcription factors, such as nuclear factor of activated T cells (NFAT), leading to the production of cytokines

Over time, the receptors’ architecture has seen substantial change and the term “generations” of CAR often refers to the number and type of intracellular signaling domains. The first generation of CARs included an activation motif (ITAM, often CD3z) and a domain with scFv to detect tumor antigens [164]. Unfortunately, these CARs were unable to provide the long-term markers of cell proliferation necessary to maintain antitumor efficacy. Additionally, CD134 (OX40), CD28, and CD137 (4-1BB) molecules were inserted into the second and third-generation CARs to increase their ability to proliferate and to be cytotoxic [169]. The following (fourth) generation of CARs is designed to release cytokines and are frequently equipped with several co-stimulatory molecules, such as CD134, CD28, or CD137, to boost their cytotoxic power against tumor cells and to stimulate the immune system (Fig. 4) [170, 171].

Furthermore, several next-generation CARs with advanced features have been developed and are presently being tested in experiments [172–174]. CAR-modified T/NK-cell therapy has demonstrated significant efficacy in the treatment of some hematological malignancies, including lymphoma, chronic lymphocytic leukemia (CLL), and acute lymphoblastic leukemia (ALL). Especially remarkable complete response rates of 70–90% were achieved in ALL patients treated with CD19–targeting CAR-T cells [175].

CAR-T/NK cell therapy for melanoma is developing rapidly, and clinical experiments using CAR-T cells are currently investigating c-MET, CD70, GD2, and VEGFR2 as melanoma-specific antigens [176, 177].

One of the promising candidates among cell types with therapeutic potential is the NKT cell, which is a subset of the innate immune system present in the circulation [178]. NKT cells play a crucial role in bridging the innate and adaptive immune systems by producing various bioactive molecules, and contribute significantly to the upregulation of the immune system and the suppression of the tumor microenvironment [179]. Moreover, enhanced cytolytic capabilities and a remarkable ability to infiltrate the TME, greatly improve their efficacy in targeting tumor cells [180, 181].

NKT cells can further stimulate other immune cells by secreting interleukin 2 (IL-2) and IL-21, resulting in the activation of NK cells and T cells. The incorporation of chimeric antigen receptors (CARs) into NKT cells enhances their cytotoxic potential, making them a potent therapeutic strategy against various types of cancer [182].

High molecular weight melanoma-associated antigen (HMW-MAA), also known as chondroitin sulfate proteoglycan 4 (CSPG4), is another significant melanoma-specific tumor antigen and is found in more than 90% of melanomas [183]. Simon et al. [184] generated functional and effective CAR-NKT cells that expressed fewer cytokines than CD8 ^+^ T cells, limiting the possibility of cytokine release syndrome, by electroporating natural killer T (NKT) cells with RNA encoding the chimeric receptor that recognizes HMW-MAA. By employing a GD2-specific target module, Mitwasi et al. [185] demonstrated in vitro specific lysis of melanoma cells expressing disialoganglioside GD2. Additionally, even after losing their transduced CARs, the intrinsic cytotoxic activity of NK CAR cells was preserved after mRNA-based receptor transfer, indicating that they might still contribute to cancer immunosurveillance. So, CAR-NKT melanoma treatment strategies, as alternatives to the current treatment methods, may represent a beneficial alternative to conventional CAR-T cells in the future development of innovative treatment techniques. Furthermore, in 2D or 3D in vitro experiments, CAR NK-92 cells effectively target CD276 (B7-H3), which is highly expressed in melanoma cells. It has been demonstrated that the immunosuppressive milieu around solid tumors adversely affects the cytotoxic capacity of NK cells. This study investigated how soluble factors released by cancer cells or cancer-associated cells, such as TGF-β, affect the effector function of CAR-mediated NK-92 cells. Overall, there was no evidence of an adverse effect on CD276-CAR NK-92 cell-mediated cytotoxicity when immunosuppressive mediators such as TGF or co-incubation with cancer-associated fibroblasts were present. The function of CD276-CAR NK-92 cells was only minimally influenced by tumor cell supernatants and prolonged hypoxic culture conditions [186].

## Conclusion

The induction of the antitumor response of the immune system depends heavily on NK cells. Although higher tumor-infiltrating NK cell content has been associated with a better prognosis in some human solid tumors, the immunosuppressive TME decreases their efficacy in favor of neoplastic growth. It is crucial to understand the mechanisms used by the TME to impair NK cell function and how they can be neutralized to create effective anti-melanoma therapeutic protocols.

NK cells are plastic, and the function of NKs in melanoma depends on the soluble chemicals in the melanoma TME and the cell-cell or tumor-cell interactions those results in dynamic phenotypic changes in infiltrating NK cells, with a gradual transition from lethal NK cells in the early stage to depleted or immunosuppressive cells in the advanced stage.

Overall, the TME is essential for NK cells to function normally and that additional research and preclinical studies in this area will probably be required to fully understand NK cell biology and reveal novel and exciting anti-melanoma therapeutic opportunities.

Many cancer immunotherapies have been developed that entail genetically altering NK cells before adoptive transfer into patients. It may be possible to modify NK cells so that they are immune to both the immunosuppressive molecules produced by the tumor and TME, as well as the metabolically constricting TME.

To develop individualized NK cell-directed therapies, it is essential to improve our understanding of how NK cells interact with metastatic melanoma cancer cells. There is an urgent need to create better preclinical models that depict the physical interactions between melanoma cells and NK cells during metastasis.

## Data Availability

Not applicable.

## Abbreviations

NK cells: Natural killer
TME: Tumor microenvironment
TGF-β: Transforming growth factor-beta
CAFs: Cancer-associated fibroblasts
VEGF: Vascular endothelial growth factor
LCs: Langerhans cells
TLRs: Toll-like receptors
PAMPs: Pathogen-associated molecular patterns
DAMPs: Damage-associated molecular patterns
MHC: Major histocompatibility complex
HMGB1: High mobility group box 1
APCs: Antigen-presenting cells
TAB: Tumor-associated B cells
FGF2: Fibroblast growth factor 2
ICIs: Immune checkpoint inhibitors
NCRs: Natural cytotoxicity receptors
NKG2D: Natural-Killer Group 2 Member D
CIML: Cytokine-induced memory-like NK cells
